# Relative Action of Anæsthetics

**Published:** 1881-05

**Authors:** 


### ARTICLE IV.

### *Relative Action of Anæsthetics.*

It is, perhaps, known to most readers that in 1877 a committee was appointed by the British Medical Association to investigate the action of anæsthetics. A complete account of their investigations from that date down to the present time is printed in the journal of the Association for December 18, 1880. It is long and thorough, giving a complete account of the various substances alleged to have anæsthetic effects, and especial investigations into ethidene dichloride, chloroform and ether. Their respective influence on the pulse-respiration ratio, on blood pressure and on pulmonary circulation, are set forth. Space does not permit us to give even an abstract of these important studies, but the *practical rules* with which the Committee concluded their report are too valuable to be omitted.

They are as follows :—

1. It is not only necessary to watch the effect of the anæsthetic upon the pulse, but it is also requisite to have regard to the respiration. We must not only take into account the danger of sudden stoppage of the respiration, but must also remember that, in the event of abnormal increase of respiratory movements, it may become essential, for the safety of the patient, to temporarily discontinue the administration.

2. Owing to the tendency of chloroform and ethidene—particularly chloroform—to reduce the blood pressure suddenly, not only during the administration of these agents, *but also after they have been stopped for some little time* (a source of serious danger,) it is necessary for the person who has charge of the administration of the drug to be on the lookout for symptoms of this occurrence, both during the time the agent is being given, and for some time after the patient has recovered from its evident effects.

3. The danger of death from stoppage of the respiratory functions, must be borne in mind in every case in which anæsthetics are given , but, of perhaps greater importance is the danger from interference with the proper action of the heart—particularly when it is remembered that, by artificial means, we can combat the former contingency. It might even be advisable, in certain cases, to introduce a tracheal tube by the mouth, so as to enable us to force air into the lungs by means similar to those adopted in experiments with animals; or, in circumstances where such a procedure was impracticable, tracheotomy might be performed, with the same object in view. Artificial respiration should be continued, even though all evidence of cardiac action has ceased.

As regards comparative danger, the three anæsthetics may be arranged in the following order : chloroform, ethidene, ether; and the ease with which the vital functions can be restored may be conversely stated, thus: the circulation is more easily re-established when its cessation is due to ether than to ethidene; and when the result of ethidene, than when chloroform has been used. The advantages which chloroform possesses over ether—in being more agreeable to the patient and more rapid in its action, in the complete insensibility produced by it, and the absence of excitement or movements during the operation—are more than counterbalanced by its additional dangers.

5. The chief dangers are: (1) sudden stoppage of the heart; (2) reduction of the blood pressure; (3) alteration of the pulse respiration ratio; (4) sudden cessation of the respiration. The danger with ether approaches from the pulmonary rather than from the cardiac side—so that, by establishing artificial respiration, we have a means of warding off death. Its advantages are to a great extent, obviated by the use of ethidene; while the dangers of chloroform are also reduced to a minimum.

Suggestions for the Safe Administration of Anæsthetics.

A suggestion to render the administration of chloroform safe, is made by Dr. A. Crombie, in the *Practitioner*, Dec., 1880. He begins the chloroform in the usual way, by inhalation, and immediately injects, with the hypodermic syringe, twenty minims of the officinal solution of muriate of morphia. This greatly prolongs the anæsthesia, so that a very small quantity of chloroform suffices to keep it up afterward, half a drachm to a drachm being usually enough to keep the patient insensible for thirty to forty-five minutes. In long and difficult operations, especially about the mouth and face, this is no small advantage; thus in removing the right upper maxilla, Dr. C. was enabled nearly to complete the operation without the further administration of chloroform from the time that the patient was declared "ready." Vomiting is rarely seen when this method is followed. Chloroform asphyxia also seems to be prevented. In 600 cases, Dr. C., has only once seen it, and in that instance some of the usual precautions had been neglected. Paralysis of the heart, too, is less apt to happen, the risk of the occurrence of this accident, and of asphyxia as well, being lessened in proportion to the smallness of the dose of the anæsthetic required to cause and reproduce the anæsthesia. It is pointed out that this method makes chloroform as safe to use as ether; and that in tropical countries, especially India, where operations have to be performed at a temperature very little below the boiling point of ether, there is practically no choice of anæsthetics.

This suggestion, however, and similar ones for combining anæsthetics with opium preparations meets a critic in Dr. D. H. Jacob, who writes to the *British Medical Journal*, in January of this year, the following warning:—

"May I utter a word of warning on the combined effects of ether and opium? Several cases have recently come to my knowledge—the details of which, I hope, will soon be published—in which death followed a short time after the administration of ether to patients under the influence of opium. In the table of deaths from ether, which you have just published, out of six cases in which death could be directly attributed to the anæsthetic, two were cases of hernia; and, therefore, most probably under the influence of opium. Whether it be a case of this kind, or morphia be given subcutaneously, in order to obtain the benefits of 'mixed anæsthetics,' ether is not without danger; and the patient should be carefully watched, after the operation, till complete awakening has taken place. In the recent volume of Billroth's *Deutsche Chirurgie*, which is devoted entirely to anæsthetics, Dr. Krappeler, from an experience of twenty-five cases, speaks strongly against the practice of mixed anæsthesia by morphia and ether; but allows that by morphia and chloroform to be open to less objection. Whether death occurs by the ether deepening the morphia narcosis, or by the morphism preventing the patient from clearing his bronchi from the secretion provoked by the ether, I will not undertake to say. It appears to me to be a very proper question for physiological experiment.

Similar warnings have been uttered before against this form of 'mixed anæsthesia;' but I do not think public attention has been called before to this explanation of deaths in hernia operation under the use of ether."

Dr. E. L. Hussey, in the London *Med. Times and Gazette*, Dec. 18, inveighs strongly against the use of the towel or napkin. Referring to a fatal case, in that country, he writes:—

"It is impossible to resist the conclusion that in many of the unfortunate cases which have been under public notice the patient lost his life through carelessness: that, in fact, he was smothered with the apparatus in which the anæsthetic was given. In this case from America we have again the story of the towel. It is the same in almost all the fatal cases; a towel, a napkin, a fold or two of lint, is used; something, in short, which of itself is a bar to the natural process of respiration. The symptoms which precede the stoppage of the pulse and the breathing are not always reported completely; perhaps they are not always observed. Artificial respiration fails to restore life."

A well made *inhaler*, he believes, should always be employed.

A combined method of administration is recommended, for its convenience and safety, by Mr. Woodside Braine, F. R. C. S., in the *British Medical Journal*, December 4. He writes:—

"The quickest, and, in my opinion, the best, method of giving ether is to administer nitrous oxide till the patient is completely anæsthetized, and then to change the face-piece without allowing the inhalation of any air whatever. During the first few respirations the larynx resents the ether-vapor, and they are somewhat jerky and spasmodic; but the larynx soon becomes accustomed to the irritant, the breathing becomes full, easy and regular, and there is a complete absence of struggling. When the struggling does occur, the movements are generally referable to dreams which the patient is having; and when he wishes to alter the position of his limbs, or to sit up, in pursuance of the nature of his dream, forcible prevention only makes him struggle more violently to accomplish his object, which, if effected in the first place, would have had no practical result, and he would have dropped back into the former recumbent position of his own accord. Should this struggling take place, no attempt should be made to hold the patient in any one position; his limbs should be allowed to move about freely, and he should be permitted to sit up, if he desire to do so—only preventing him from pulling off the face-piece.

But, in addition to the trouble saved by not having to hold a patient, the risk of a fatal result, especially when administering chloroform, is much less; for, if the patient be allowed to move freely, the heart has comparatively little work to do, compared with the strain on it which exists when it has to drive a column of blood through vessels which have rigid muscles on each side of them; and, again, this very struggling increases the venous flow, and tends to gorge the heart with venous blood.—*Med. and Surg. Reporter.*

